# Artificial intelligence guided imaging as a tool to fill gaps in health care delivery

**DOI:** 10.1038/s41746-025-01613-2

**Published:** 2025-05-05

**Authors:** Ben Li, Elizabeth J. Enichen, Kimia Heydari, Joseph C. Kvedar

**Affiliations:** 1 Division of Vascular Surgery, University of Toronto, Toronto, ON Canada; 2 Temerty Centre for Artificial Intelligence Research and Education in Medicine, University of Toronto, Toronto, ON Canada; 3 Harvard Medical School, Boston, MA USA

**Keywords:** Thromboembolism, Diagnosis, Ultrasonography

## Abstract

Deep vein thrombosis (DVT) causes significant morbidity/mortality and timely diagnosis often via ultrasound is critical. However, the shortage of trained ultrasound providers has been an ongoing challenge. Recently, Speranza and colleagues (2025) demonstrated that an artificial intelligence (AI) guided ultrasound system used by non-ultrasound-trained nurses with remote clinician review can achieve sensitivities of 90–98% and specificities of 74–100% for diagnosing DVT. This study highlights the potential for AI guided imaging to address important gaps in health care delivery.

## Introduction

Deep vein thrombosis (DVT) involves blood clot formation primarily within major leg veins, which can travel to the lungs, leading to life-threatening pulmonary embolism^1^. Therefore, prompt diagnosis and management of DVT is critical^1^. Although invasive venography is the gold standard, the most common way to diagnose DVT is through ultrasound, whereby a technician uses an ultrasound probe to compress the vein(s) with suspected DVT^2^. A non-compressible vein is diagnostic for DVT, and treatment is generally started with blood thinners^2^.

Most clinicians are not trained to perform DVT ultrasounds, and therefore, ultrasound technicians are necessary^3^; however, they are not always available^4^. A survey of 79 US teaching hospitals showed that only 24% had ultrasound technicians on the premises after hours between 6pm and 8am, when patients can present acutely with DVT’s^5^. Although other imaging modalities can be used to diagnose DVT, they are generally more expensive and can be less accurate than ultrasound^6^. Empiric treatment of suspected DVT’s with blood thinners prior to imaging confirmation carries considerable risks, including life-threatening bleeding^7^. Therefore, there is an important need to address this gap in diagnostic care.

Artificial intelligence (AI) has the potential to guide non-ultrasound-trained providers in performing DVT ultrasounds with sufficient diagnostic quality^8^. Recently, Speranza and colleagues (2025) assessed the use of AI-guided ultrasound by non-ultrasound-trained nurses to diagnose DVT^9^. In this article, we highlight key findings from Speranza et al.’s study and discuss the potential for AI-guided imaging to fill important gaps in healthcare delivery.

## An AI-guided ultrasound system

Speranza and colleagues evaluated the *ThinkSono Guidance System* (ThinkSono GmbH; https://thinksono.com/), an AI system that guides non-ultrasound-trained providers in performing DVT ultrasounds^9^. The system consists of a smartphone application and a portable ultrasound device^9^. The software guides the operator in using the ultrasound probe to compress the common femoral and popliteal veins (the highest probability sites for identifying DVT’s) several times^1,9^. Each time, the operator receives feedback on the compression location, timing, and positioning^9^. The entire process generally takes less than 10 minutes^9^. Once the software confirms the acquisition of appropriate images, they are uploaded to a secure cloud dashboard and reviewed by a remote medical imaging specialist to diagnose DVT^9^.

## Key findings by Speranza and colleagues

Speranza and colleagues conducted a retrospective analysis of data from a multicenter, prospective, double-blinded pilot study designed to evaluate the accuracy of AI-guided ultrasound imaging for DVT diagnosis^9^. The study was conducted across 11 UK hospitals and included adults with symptoms suggestive of DVT who required a diagnostic ultrasound scan^9^. Overall, 381 patients between 2021–2023 were included, all of whom underwent both an AI-guided ultrasound scan by a non-ultrasound-trained nurse and an expert sonographer-performed ultrasound scan^9^. Each AI-guided scan was reviewed remotely by a blinded radiologist or point-of-care ultrasound (POCUS) certified emergency physician^9^. The DVT diagnosis made by the remote clinician using images from the AI-guided ultrasound was compared to the ground truth, defined as the DVT diagnosis obtained from the local imaging specialist report based on the expert sonographer scan, which is the standard of care^9^. In the study, 80% of AI-guided scans received an American College of Emergency Physicians (ACEP) image quality score ≥ 3, which constitutes adequate image quality to diagnose DVT^9^. The AI-guided ultrasound system achieved sensitivities of 90-98% and specificities of 74–100% for diagnosing DVT^9^, which can be considered a good to excellent diagnostic test as defined by Power and colleagues (2013)^10^.

## Implications of an AI-guided ultrasound system to diagnose DVT

Speranza and colleagues demonstrated that an AI-guided ultrasound system used by non-ultrasound-trained nurses with remote clinician review can achieve relatively high sensitivity and specificity for diagnosing DVT^9^. Since nurses are generally available 24/7 in hospital settings, this technology has the potential for important clinical impact^11^. Given our aging and increasingly sedentary society with a rising incidence of DVT’s in combination with constrained health care budgets and worker shortages, the lack of available ultrasound technicians to perform DVT scans will be an escalating challenge^12,13^. This technology could temporarily address the shortage of trained ultrasound providers while long-term solutions are implemented, including increasing health care funding, worker capacity, and infrastructure^14^.

The other potential application of AI-guided ultrasound systems is the ability to reduce operator variability, even amongst expert sonographers^15^. Ultrasound scanning can vary significantly between providers based on the specific location scanned, probe angle, and amount of pressure applied, among other factors^16^. This variability can lead to differences in image quality and inaccurate diagnoses^16^. AI-guided systems can make the scanning process more systematic through real-time instructions and feedback, thereby reducing variability amongst operators^15^.

## Limitations

Although the performance metrics of the AI-guided ultrasound system for diagnosing DVT are satisfactory, there is room for improvement^9^. Based on the study findings, 20% of AI-guided scans would be of insufficient quality, and a non-negligible proportion of DVT diagnoses would be incorrect, leading to inappropriate management^9^. Therefore, additional refinement of the technology to improve image quality and diagnostic accuracy may further increase its clinical utility^9^. Additionally, this was a retrospective analysis of data from a pilot study^9^. Prospective validation of this technology on larger cohorts is needed to support clinical implementation.

## Conclusions

Through a retrospective analysis of a multicenter, prospective, double-blinded pilot study, Speranza and colleagues (2025) demonstrated that an AI-guided ultrasound system used by non-ultrasound-trained nurses with remote clinician review can achieve relatively high sensitivity and specificity for diagnosing DVT^9^. This study highlights the potential for AI tools to fill important gaps in healthcare delivery by supporting timely DVT diagnosis when ultrasound technicians are not available^9^. Importantly, AI-guided imaging can be applied to many other areas of health care to standardize imaging protocols, reduce operator variability, and potentially improve diagnostic accuracy, ultimately improving patient care.

## Data Availability

No datasets were generated or analyzed during the current study.
